# Level of respiratory protection for healthcare workers caring for coronavirus disease 2019 (COVID-19) patients: A survey of hospital epidemiologists

**DOI:** 10.1017/ice.2021.74

**Published:** 2021-02-19

**Authors:** Winston L. McCormick, Michael P. Koster, Geetika N. Sood, Leonard A. Mermel

**Affiliations:** 1 Warren Alpert Medical School of Brown University, Providence, Rhode Island; 2 Division of Infectious Diseases, Department of Epidemiology & Infection Control, Rhode Island Hospital, Providence, Rhode Island; 3 Division of Infectious Diseases, Department of Epidemiology & Infection Control, Johns Hopkins Bayview Medical Center, Baltimore, Maryland

*To the Editor—*Appropriate personal protective equipment (PPE) for healthcare workers (HCWs) caring for patients with coronavirus disease 2019 (COVID-19) has not yet been completely elucidated nor universally standardized. In areas where severe acute respiratory coronavirus virus 2 (SARS-CoV-2) community transmission is moderate or substantial, the Centers for Disease Control and Prevention (CDC) recommends at least N95 respirator protection, eye protection, gowns, and gloves for patient-facing HCWs. The lack of a centralized national process for the acquisition and stockpiling of PPE has resulted in severe shortages across the United States, forcing competition among hospital systems and leading to extreme measures to procure PPE for HCWs.^1,9^

Infection control departments have developed triage plans for PPE use in the face of uncertain and insufficient supplies in an effort to mitigate COVID-19 risk among HCWs and hospitalized patients. Recommendations were often based on intuition and extrapolation of data from other respiratory viral infections, especially as it relates to respiratory protection. Studies of influenza and seasonal coronavirus virus have revealed that surgical masks are not inferior to N95 respirators for source control.^2,4,5,7,8^ Studies of SARS-CoV-1 have demonstrated no significant difference between surgical masks and N95 respirators with respect to protection of exposed HCWs.^3,7^ Surgical masks for patient source control and worn by HCWs, especially coupled with HCW use of face shields, protect HCWs involved in routine COVID-19 patient care who are not participating in aerosol-generating procedures (AGPs).^5,6^ Given that N95 respirators are superior to surgical masks in protecting the user from inhalation of small-particle aerosols, their use has been typically reserved for AGPs.^2,4,5^

## Methods

On October 6, 2020, we performed an online survey of infection control leaders regarding their respiratory PPE policies for general routine patient care, general care of patients with confirmed or suspected COVID-19, and care for patients undergoing AGPs. In addition, we asked whether routine retesting of admitted patients was performed at some interval after admission.

## Results

Of the 56 hospitals or health systems, 29 responded (52% response rate); 5 were with the Department of Veterans’ Affairs (VA) (Table 1). The sites were located in or included Providence, Rhode Island; Omaha, Nebraska; Denver, Colorado; Charlottesville, Virginia; Charlotte, North Carolina; Royal Oak, Michigan; Chicago, Illinois; Boston, Massachusetts; Ann Arbor, Michigan; St. Louis, Missouri; Salt Lake City, Utah; Baltimore, Maryland; Pittsburgh, Ohio; Cleveland, Ohio; San Francisco, California; New York City, New York; Iowa City, Iowa; Dallas, Texas; Buffalo, New York; San Antonio, Texas; Montreal, Quebec, Canada; the VISN-10 network of VA hospitals; as well as VA systems in Texas, Connecticut, and New York.

**Table 1.** Personal Protective Equipment (PPE) Use Among Respondent Hospitals and Hospital Systems

Hospital	Respiratory PPE Used While Caring for Patients With Confirmed or Suspected COVID-19	Respiratory PPE for General Patient Care	Respiratory PPE Use for AGPs	Retest COVID-19 Patients
Non-VA hospitals or health systems (n = 24)	N95 respirator (n = 15) Surgical mask (n = 9)	N95 respirator (n = 2) Surgical mask (n = 20)	N95 respirator, regardless of SARS-CoV-2 status (n = 20) Surgical mask if known negative SARS-CoV-2 test (n = 2)	Yes, if symptomatic (n = 10) Yes, if before procedures (n = 11) Yes, every 5 d (n = 1) Yes, on day 5 if from high risk setting (n = 1) Yes, every 3 d if in multiroom or behavioral health unit (n = 1)
VA hospitals or health systems (n = 5)	N95 respirator (n = 4) Surgical mask (n = 1)	N95 respirator (n = 0) Surgical mask (n = 4)	N95 respirator, regardless of SARS-CoV-2 status (n = 2) Surgical mask if known negative SARS-CoV-2 test (n = 1)	Yes, if symptomatic (n = 3) Yes, if before procedures (n = 1)

Note: COVID-19, coronavirus disease 2019; SARS-CoV-2, severe acute respiratory coronavirus virus 2; APGs, aerosol-generating procedures.

Variability in respiratory PPE was reported in the care of patients with proven or suspected COVID-19. Overall, 63% of non-VA hospitals or health systems used N95 respirators when caring for such patients. There was greater consistency among VA respondents, with 80% using N95 respirators when caring for patients with proven or suspected COVID-19. Not all hospitals or hospital systems used N95 respirators during AGPs. Some (33% of VA respondents and 8% of non-VA respondents), adopted a strategy for use of surgical masks during AGPs in patients negative for SARS-CoV-2. Notably, 27 of 29 respondents to a question about eye protection affirmed such use when caring for patients with COVID-19. There was no consistency in the recommendations reported regarding the retesting of patients.

## Discussion

Definitive data are still lacking regarding the necessary and sufficient PPE when caring for patients with COVID-19, during AGPs, and while providing routine patient care in a pandemic fraught with high asymptomatic case loads. As such, the variability we found in PPE use suggests that more data are required to fully understand SARS-CoV-2 transmission routes to inform appropriate universal PPE recommendations. A limitation of this survey is that we did not ask what PPE would be recommended if resource limitations did not exist. Every hospital and hospital system had to develop local guidance based on local resources, and our survey does not capture this. We do know that supplies have yet to meet demands.

Transmission risk is also dependent on source control of suspected and asymptomatic patients, and unfortunately, we did not ask about this important infection prevention strategy in our survey. We did ask about retesting of admitted patients as an infection control practice, and we found wide variability, likely reflecting both limitations of testing resources as well as lack of data for utility of this practice.

December 2020 ushered in a new phase of the COVID-19 pandemic. Two vaccines were given FDA emergency use authorization, and the new, more highly transmissible B117 variant was discovered on American shores.^10^ The B117 strain appears to be more contagious, and while we wait on official CDC and World Health Organization (WHO) guidance, current COVID-19 guidelines for PPE and vaccinations appear to be sufficient.^10^ Hospital epidemiologists will need to continue to assimilate evolving evidence to best protect HCWs from contracting COVID-19 in the face of a continuing pandemic and ongoing supply-chain shortages. Further elucidation of transmission dynamics will assist infection control departments facing ongoing PPE shortages and emerging SARS-CoV-2 mutant strains of this RNA virus.

In conclusion, most hospitals and hospital systems responding to our survey use N95 respirators when caring for patients with proven or suspected COVID-19 and when aerosol-generating procedures are performed. While we await WHO and CDC guidance on how vaccines and highly transmissible mutant strains will affect PPE recommendations, the 2 opposing forces will likely draw out the need for enhanced PPE in healthcare settings.
